# Mixed Reality Combined with Three‐Dimensional Printing Technology in Total Hip Arthroplasty: An Updated Review with a Preliminary Case Presentation

**DOI:** 10.1111/os.12537

**Published:** 2019-10-29

**Authors:** Peng‐fei Lei, Shi‐long Su, Ling‐yu Kong, Cheng‐gong Wang, Da Zhong, Yi‐he Hu

**Affiliations:** ^1^ Department of Orthopaedics Xiangya Hospital, Central South University Changsha China; ^2^ Department of Radiology Xiangya Hospital, Central South University Changsha China

**Keywords:** Mixed reality technology, Registration, Three‐dimensional printing technology, Total hip arthroplasty

## Abstract

Three‐dimensional (3D) printing technology, virtual reality, and augmented reality technology have been used to help surgeons to complete complex total hip arthroplasty, while their respective shortcomings limit their further application. With the development of technology, mixed reality (MR) technology has been applied to improve the success rate of complicated hip arthroplasty because of its unique advantages. We presented a case of a 59‐year‐old man with an intertrochanteric fracture in the left femur, who had received a prior left hip fusion. After admission to our hospital, a left total hip arthroplasty was performed on the patient using a combination of MR technology and 3D printing technology. Before surgery, 3D reconstruction of a certain bony landmark exposed in the surgical area was first performed. Then a veneer part was designed according to the bony landmark and connected to a reference registration landmark outside the body through a connecting rod. After that, the series of parts were made into a holistic reference registration instrument using 3D printing technology, and the patient's data for bone and surrounding tissue, along with digital 3D information of the reference registration instrument, were imported into the head‐mounted display (HMD). During the operation, the disinfected reference registration instrument was installed on the selected bony landmark, and then the automatic real‐time registration was realized by HMD through recognizing the registration landmark on the reference registration instrument, whereby the patient's virtual bone and other anatomical structures were quickly and accurately superimposed on the real body of the patient. To the best of our knowledge, this is the first report to use MR combined with 3D printing technology in total hip arthroplasty.

## Introduction

Artificial joint replacement is standard practice for relieving pain and restoring function in cases of damaged joints^1^, ^2^. However, it is still a great challenge for orthopaedic surgeons to deal with complicated hip joint disease caused by congenital disease, rheumatic disease, trauma, infection, and other serious diseases^3^, ^4^. Orthopaedic surgeons have to draw on their own experience to perform such complicated hip replacement surgeries, with the help of general imaging examinations, such as plain films, CT, and MRI^5^, ^6^. For these complicated total hip arthroplasties, it is often necessary for the surgeon to have a wealth of surgical experience. Therefore, are many potential problems for inexperienced surgeons when dealing with a complicated hip arthroplasty. For example, it is difficult for young surgeons to judge the orientation of the osteotomy, the position of the true acetabulum, the installation position of the prosthesis, and the exposure of the anatomical safe range. These difficulties often lead to operation complications, such as prolonged operation time, larger surgical incision, and more bleeding^3^, ^4^, ^7^, which can result in poor prognosis.

Since 2010, new technologies have been applied to help perform complicated artificial joint replacement surgeries. For example, 3D printing technology can be used to fabricate the 3D solid model that mimics the structure of the dysfunctional bone to help surgeons have a more intuitive understanding of the lesion area, and develop a rational surgical plan^8^, ^9^. However, 3D printing technology cannot reveal the structure of soft tissue, blood vessels, and nerves in a lesion area. Surgical navigation systems^10^ and surgical robots^11^ provide strong support for the implementation of precision surgery, but the high associated costs, the lack of force feedback, and the tedious training process limit their use.

Since 2012, the emergence of mixed reality (MR) technology has provided a new option to simultaneously view virtual 3D images of bones, peripheral nerves, and vascular tissues in lesion areas, helping surgeons understand the condition of the surgical field in real time^12^. However, at present, the unsatisfied accuracy of registration in MR technology is an urgent problem yet to be resolved^13^. After reading the literature, we found that some researchers have designed 3D printing guide plates to assist orthopedic surgery with 3D printing technology, and verified that they have good clinical value.^14^, ^15^, ^16^ Herein, we have creatively combined 3D printing technology and MR technology, with the “volume amplification method,” providing a new concept for the accurate registration of MR technology. To the best of our knowledge, this is the first report that applies this novel technology, which combines 3D printing technology with MR technology to complete a complicated total hip replacement.

### *Case Presentation*

**Figure 1:**
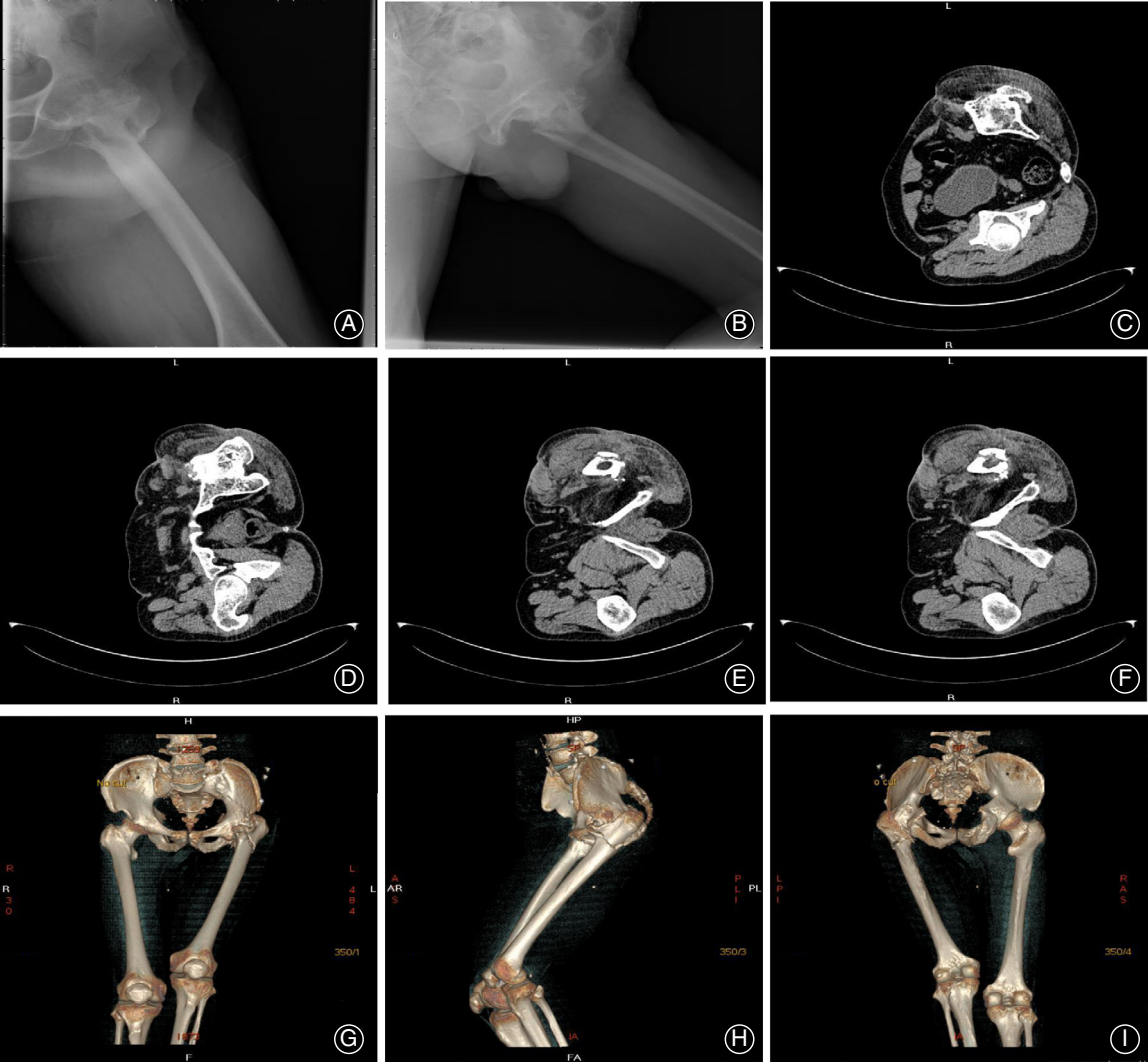
Preoperative imaging examination of the hip for the patient: (A) Anterior radiograph of the left hip joint; (B) lateral radiograph of the left hip joint; (C, D) preoperative CT images showing left hip fusion; (E, F) preoperative CT showing an intertrochanteric fracture of the left femur; and (G‐I) CT three‐dimensional reconstruction of the pelvis and femur.

**Figure 2:**
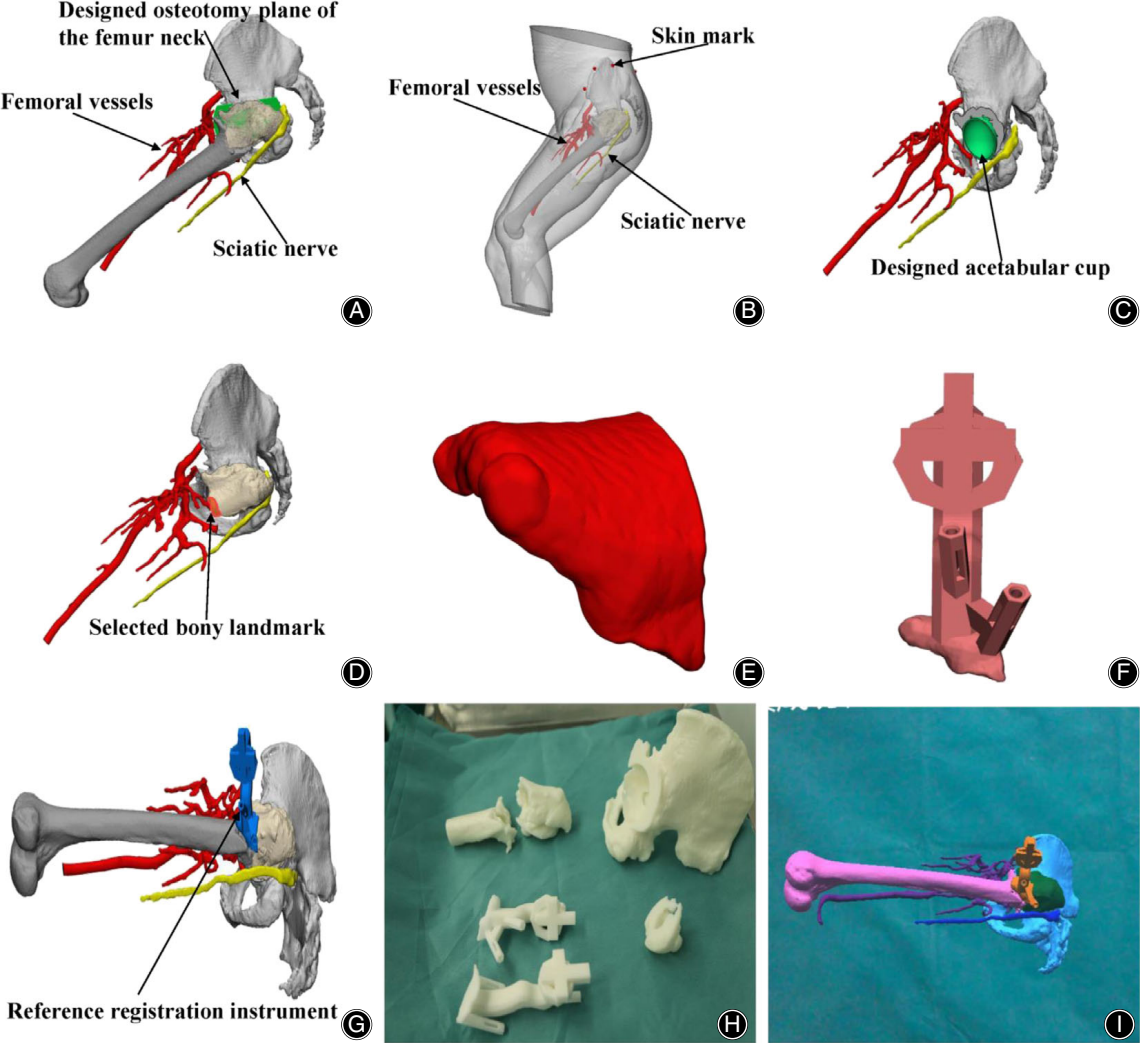
Preoperative operative design for the patient: (A) The designed osteotomy plane (cyan part) of the femur neck was very close to the sciatic nerve (yellow part) and the femoral vessels (red part). (B) The three‐dimensional (3D) fracture digital model is presented. (C) The location and size of the designed acetabular cup (cyan part) are presented. (D) The selected bony landmark (orange part) is presented. (E) The personalized veneer part simulation. (F) The reference registration instrument simulation. (G) The reference registration instrument (blue) was installed on the selected bony landmark. (H) Sterilized 3D printed model. (I) The3D image was displayed using a head‐mounted display (HMD).

A 59‐year‐old male patient was admitted to our hospital on 17 September 2018, complaining of left hip pain accompanied with movement disorder lasting for 2 days. X‐ray and CT images showed an intertrochanteric fracture of the left femur and prior left hip fusion (Fig. 1). The left hip fusion had been performed to treat septic arthritis of the left hip more than 20 years ago. A physical examination prior to treatment revealed that the left hip joint of the patient was in a 60° flexion contracture deformity and a 90° external rotation deformity. The range of joint motion was 60°‐120° of flexion, 0° of extension, 0‐20° of abduction, and 0°‐10° of adduction. The left lower limb was 20 cm shorter than the right lower limb. The Harris score was 30.9 points. We planned to perform left total hip arthroplasty to address the left intertrochanteric fracture and hip fusion. Preoperative 3D model reconstruction (Fig. 2A) showed that the designed osteotomy plane of the femur neck was very close to the sciatic nerve and the femoral vessels. The sciatic nerve and the femoral vessels could not be well protected if the traditional surgery was performed for the patient. As such, we decided to use MR technology combined with 3D printing to help with the surgery.

Before surgery, 3D structural data of the surgical area was collected using high‐resolution CT (Philips, Eindhoven, Netherlands) and MRI (Siemens, Berlin, Germany) plain scans. CT and MRI data (DICOM format) for the surgical area were imported into the computer and reconstructed using Mimics 19.0 software (Materialize, Leuven, Belgium). Different colors were used to distinguish different structures to obtain a “3D fracture digital model” (Fig. 2B). The patient's condition was analyzed and a surgical plan was developed, including the osteotomy plane (Fig. 2A) and the location and size of the acetabular cup installation (Fig. 2C). A 3D printing reference registration instrument was established as follows. First, according to the surgical requirements, the bony landmark to potentially expose during the operation was simulated and marked on the computer (Fig. 2D), and then a personalized veneer part for the selected bony landmark was designed (Fig. 2E). Subsequently, the reference landmarks were designed as the registration landmarks outside the incision of the surgical field, and one rod was designed to connect the veneer part with the registration landmarks. Finally, the veneer part, the connecting rod, and the reference registration landmarks were combined into a whole (Fig. 2F,G), by using the 3D printing method. After the engineer's measurement, accord with the preoperative design, the assembled instrument was sterilized for use (Fig. 2H). Thereafter, the digital 3D data of the surgical area anatomy and the reference registration instrument mounted on it was input into the head‐mounted display (HMD) (Microsoft Corporation, Redmond, WA, USA) through a personal computer (Lenovo Group, Beijing, China) and opened with pre‐designed dedicated automatic matching software (Fig. 2I).

**Figure 3:**
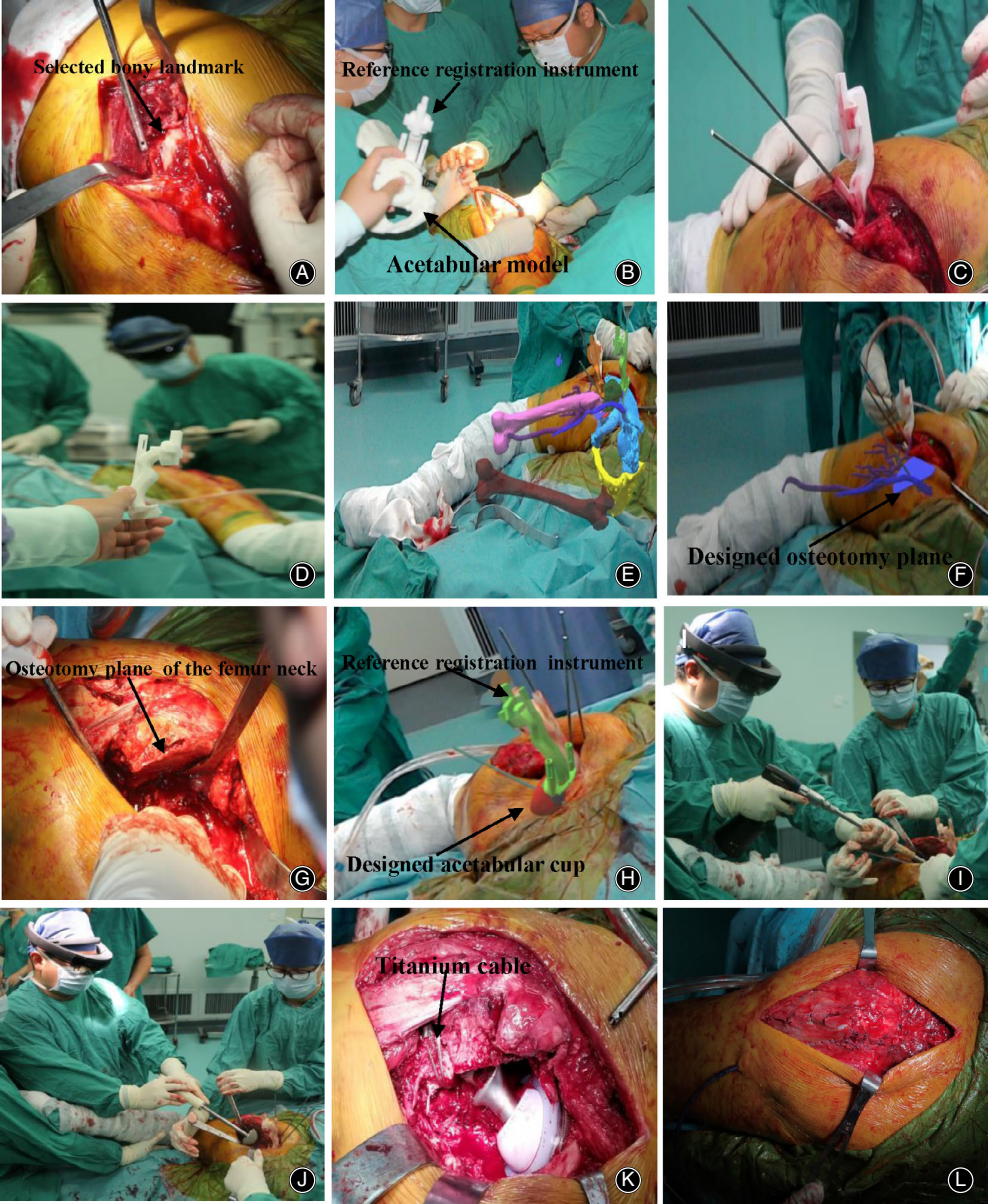
The surgical procedure and intraoperative findings: (A) Exposing the selected bony landmark according to the surgical plan; (B, C) under the assistance of the engineer, the reference registration instrument was installed on the selected bony landmark with two K‐wires; (D) registration under the assistance of the engineer; (E) the three‐dimensional virtual structures were displayed in the patient's body; (F, G) osteotomy under the guidance of the designed osteotomy plane (blue plane); (H, I) using the acetabular reamer to grind according to the designed acetabular cup (red); (J) inserting the cementless acetabular cup according to the designed acetabular cup; (K) prosthesis installment; and (L) incision suture layer by layer.

The surgery was performed through a direct posterior–lateral approach. After anesthesia through lumbar plexus and sciatic nerve block, the patient was placed in a right lateral position, before disinfection and draping. An approximate 12‐cm posterolateral incision was made on the left hip, and the epidermis and subcutaneous tissue of the surgical area were dissected to expose the selected bony landmark according to the surgical plan (Fig. 3A). With the assistance of an engineer, the personalized veneer part of the reference registration instrument was attached to the bony landmark and two K‐wires with an appropriate diameter were placed and installed in the bone through the plate hole (Fig. 3B,C). The surgeon wore the HMD and opened the automatic registration software after adjustment. With the assistance of the engineer, the reference landmarks on the reference registration instrument were used as the registration landmarks to perform automatic registration, and the anatomical structure of the surgical area was virtualized on the holographic computer and overlapped the patient himself (Fig. 3D,E). The placement of the acetabular was performed under the guidance of MR. First, the osteotomy was performed according to the preoperative design of the osteotomy plane (Fig. 3F,G). Second, in the preoperative design of the acetabulum cup position, the acetabular reamer with a diameter of 38 mm was used to grind to a diameter of 53 mm (Fig. 3H,I), and then a 54 mm diameter cementless acetabular cup (Pinnacle, DePuy Synthes, USA) was inserted (Fig. 3J) and reinforced with two acetabular screws. This process was carried out while the 3D virtual structures that were designed before surgery were displayed in the patient's body. For the conventional proximal femoral procedure, first, the femoral medullary cavity was opened with a rectangular osteotome. Then, the medullary cavity was enlarged to the size of 10.5 with the medullary cavity file. The cementless straight femoral shaft prosthesis with a size of 10.5 was installed with a short neck ceramic head with a diameter of 36 mm. Next, two titanium cables were used to fix the large rotor bone block on the femoral shaft. Finally, the hip joint was reset and checked for its motion, followed by the installment of the prosthesis (Fig. 3K). A drainage tube was placed deep into the surgical area, with suturing done layer by layer (Fig.3L). The operation lasted for 4.5 h, and there was 700 mL of intraoperative bleeding.

**Figure 4:**
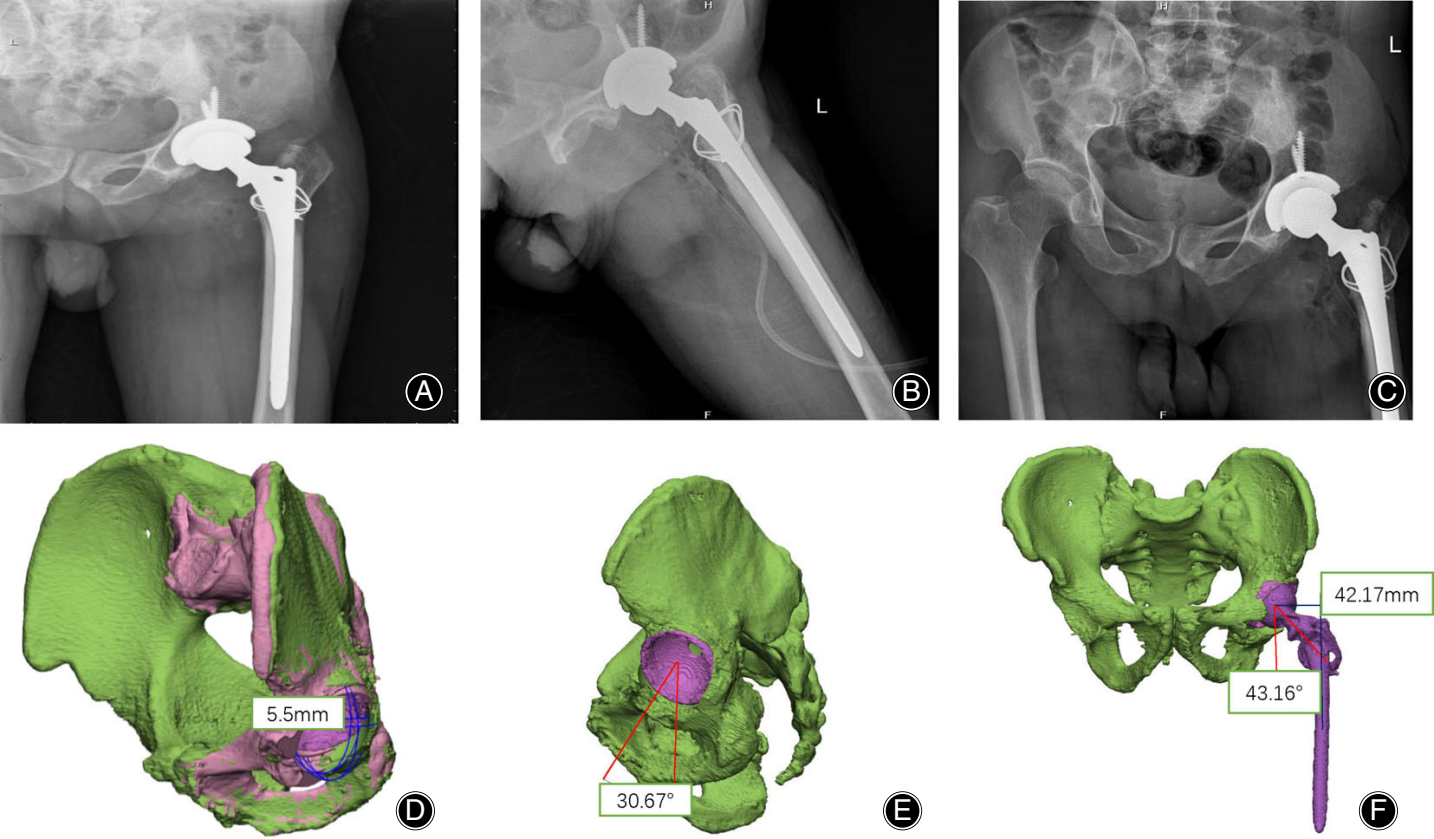
Postoperative imaging examination of the left hip joint: (A) Anterior, (B) lateral and (C) pelvis anteroposteriorly. (D) The distance between the preoperative design rotation center and the postoperative rotation center was 5.5 mm. (E) The postoperative anteversion angle was 30.67°. (F) The postoperative abduction angle was 43.16° and the femoral offset was 42.17 mm.

After surgery, the patient was treated with cefoxitin to prevent infection. X‐ray examination was performed on the second day after surgery, revealing that the position of the prosthesis was installed satisfactorily (Fig. 4A‐C). The postoperative CT data were imported into the computer and reconstructed using Mimisc19.0 software to obtain the “postoperative 3D digital model,” and compared with the preoperative data. The distance between the preoperative design rotation center and the postoperative rotation center was 5.5 mm (Fig. 4D). Meanwhile, the anteversion angle was 25° and the abduction angle was 40° in the preoperative design, while postoperative examination revealed that the anteversion angle was 30.67° and the abduction angle was 43.16°, with a femoral offset of 42.17 mm (Fig. 4E,F). The range of motion for the hip joint was within the normal range. The drainage tube was pulled out on the fourth day after surgery, and the patient began to move on the ground with the walking aid on the seventh day after surgery, exhibiting a good recovery. The patient was discharged 29 days after admission without obvious surgical complications.

## Discussion

Since 2010, digital medical technology, especially 3D printing technology, and holographic digital technology represented by MR technology have continued to be developed and put into practice. MR technology establishes an interactive feedback information loop among the virtual world, the real world, and the user by introducing real scene information in the virtual environment to enhance the realism of the user experience. It is the key element to achieving real‐time interaction among the real world, virtual models, and users.

Mixed reality technology has been applied in many fields of medicine. For medical education, it provides medical students with a more intuitive understanding of anatomical structures, as well as being useful for various inspection operations and surgical simulation exercises^17^, ^18^. In the field of neuroscience and psychiatry, MR‐based behavior rehabilitation of patients experiencing various types of phobias can be performed through biofeedback and real‐time modification of objects in the virtual environment according to the patients' interactions^19^. In addition, Zhu *et al*.^20^ used MR technology to provide accurate guidance for percutaneous nephrolithotomy. At the same time, MR technology has been applied in breast tumor resection^21^, chest wall tumor resection and reconstruction^22^, liver surgery^23^, complex cervical fracture surgery^24^, and artificial cervical disc replacement^25^.

The application of MR technology in the medical field has shown good prospects. From the case in the present study, we concluded that there are many advantages of the combination of MR technology and operation over traditional surgical methods. First, before surgery, we can have a more intuitive understanding of the lesion area, design the surgical plan and conduct the surgical simulation, and accurately predict how to safely osteotomize, how to place the prosthesis and how to deal with the lesion, which could protect the normal tissue, such as peripheral blood vessels and nerves. We can predict potential problems during the operation in advance through MR technology, whereby we can develop a personalized operation plan for the patient. Second, communications about the operation plan between doctor and patient would become easier and smoother because the patient can understand the operation plan more intuitively and have a deeper understanding of the risk of surgery through the MR technology. Third, the MR technology can present a virtual 3D image of the bones, peripheral nerves, and vascular tissue for the patient, helping the surgeon understand the condition of the surgical field in real time, and, therefore, perform accurate and safe surgery.

As an emerging technology, there are some yet unresolved problems. For example, the registration problem of MR technology is one of our preoperative concerns. There are two traditional ways to achieve better registration of MR technology. One is manual registration based on the existing structure^13^, and the other is surface reconstruction registration based on an artificial reference implanted into the human body^25^, ^26^. However, the effects of these two registration methods are not satisfactory and sometimes the two methods cannot meet the intraoperative requirements. First, the soft tissues around the bone could affect the registration and the position in the operation will be inconsistent with the preoperative marked position. Similarly, the structure of the surgical area as the registration landmark would move, which will cause a large error in registration^23^. Second, it remains a problem for surgeons to fully expose the bony landmark during operation, because the position is usually deep. Therefore, it is very difficult to complete automatic registration with a small part of the bony landmark as a registration landmark. Third, during the process of surface reconstruction registration, at least three landmarks should be positioned for precise registration. However, because the surgical field is small, placing more landmarks using traditional methods would reduce the accuracy of registration^25^.

To resolve the problem, we have creatively combined 3D printing technology with MR technology and proposed a new solution called the “volume amplification method.” On a relatively constant position of the bone, we select a bony landmark and design a reference registration instrument using 3D printing technology. The registration landmarks on the reference registration instrument are identified *in vitro* to achieve registration *in vitro*, thereby enabling registration of the entire surgical field. Through this case, the feasibility and effectiveness of the method were verified. In addition, this method would require more clinical cases to prove its validity. We believe that this method would have wide application in arthroplasty and promote the application of MR technology in clinical medicine.

In this report, the patient's hip joints were fused, and the normal anatomical structure around the hip joint could not be identified, and, therefore, could not provide a reference. Therefore, how to determine the osteotomy surface and the position of the acetabular cup has become an issue for this operation. At the same time, preoperative imaging data and 3D reconstruction images showed that the sciatic nerve of the patient was very close to the surgical area and was vulnerable to injury. Traditional surgery relies on preoperative 2D images to make the preoperative surgery plan, with operation process relying on the doctor's surgical experience and anatomical knowledge.

However, the above problems can be solved using MR technology combined with 3D printing technology. First, the preoperative 3D anatomical structure images could overcome the shortcomings of 2D data, display the lesions more intuitively and stereoscopically, and determine the osteotomy plane and acetabular cup placement on the virtual anatomical images. Second, after registration, the virtual image was combined with the human body, which enabled real‐time observation of the position of the important anatomical structures hidden in the sciatic nerve and avoided injuring the sciatic nerve. Then, the acetabular cup prosthesis was placed in the selected position according to the guidance of the virtual acetabular cup. It enabled the visual stereo guidance of virtual 3D images in every key step. Postoperative anatomical 3D reconstruction of the rotation center, and anteversion and abduction angles showed minimal errors compared with preoperative surgical planning. This is beyond the reach of traditional surgery. The entire operation lasted 4.5 hours, similar to the average time reported in the literature for such complex operations^27^. Assessing the whole process, MR‐assisted surgery achieved more precise individualized surgery without significantly increasing the operation time.

To the best of our knowledge, we completed the first total hip arthroplasty guided by MR technology combined with 3D printing technology. We proposed a new method for real‐time automatic registration of MR technology combined with 3D printing technology, which provides favorable conditions for better application of MR technology in clinical medicine.
